# Public Discourse and Sentiment Toward Dementia on Chinese Social Media: Machine Learning Analysis of Weibo Posts

**DOI:** 10.2196/39805

**Published:** 2022-09-02

**Authors:** Dexia Kong, Anfan Chen, Jingwen Zhang, Xiaoling Xiang, W Q Vivian Lou, Timothy Kwok, Bei Wu

**Affiliations:** 1 Department of Social Work Chinese University of Hong Kong Hong Kong Hong Kong; 2 School of Journalism and Communication The Chinese University of Hong Kong Hong Kong Hong Kong; 3 Department of Communication University of California, Davis Davis, CA United States; 4 Department of Public Health Sciences University of California, Davis Davis, CA United States; 5 School of Social Work University of Michigan Ann Arbor, MI United States; 6 Department of Social Work & Social Administration Sau Po Centre on Ageing University of Hong Kong Hong Kong Hong Kong; 7 Faculty of Medicine Chinese University of Hong Kong Hong Kong Hong Kong; 8 Rory Meyers College of Nursing New York University New York, NY United States

**Keywords:** dementia, public discourse, sentiment, Weibo, social media, machine learning, infodemiology, aging, elderly population, content analysis, topic modeling, thematic analysis, social support, sentiment analysis, public discourse

## Abstract

**Background:**

Dementia is a global public health priority due to rapid growth of the aging population. As China has the world’s largest population with dementia, this debilitating disease has created tremendous challenges for older adults, family caregivers, and health care systems on the mainland nationwide. However, public awareness and knowledge of the disease remain limited in Chinese society.

**Objective:**

This study examines online public discourse and sentiment toward dementia among the Chinese public on a leading Chinese social media platform Weibo. Specifically, this study aims to (1) assess and examine public discourse and sentiment toward dementia among the Chinese public, (2) determine the extent to which dementia-related discourse and sentiment vary among different user groups (ie, government, journalists/news media, scientists/experts, and the general public), and (3) characterize temporal trends in public discourse and sentiment toward dementia among different user groups in China over the past decade.

**Methods:**

In total, 983,039 original dementia-related posts published by 347,599 unique users between 2010 and 2021, together with their user information, were analyzed. Machine learning analytical techniques, including topic modeling, sentiment analysis, and semantic network analyses, were used to identify salient themes/topics and their variations across different user groups (ie, government, journalists/news media, scientists/experts, and the general public).

**Results:**

Topic modeling results revealed that symptoms, prevention, and social support are the most prevalent dementia-related themes on Weibo. Posts about dementia policy/advocacy have been increasing in volume since 2018. Raising awareness is the least discussed topic over time. Sentiment analysis indicated that Weibo users generally attach negative attitudes/emotions to dementia, with the general public holding a more negative attitude than other user groups.

**Conclusions:**

Overall, dementia has received greater public attention on social media since 2018. In particular, discussions related to dementia advocacy and policy are gaining momentum in China. However, disparaging language is still used to describe dementia in China; therefore, a nationwide initiative is needed to alter the public discourse on dementia. The results contribute to previous research by providing a macrolevel understanding of the Chinese public’s discourse and attitudes toward dementia, which is essential for building national education and policy initiatives to create a dementia-friendly society. Our findings indicate that dementia is associated with negative sentiments, and symptoms and prevention dominate public discourse. The development of strategies to address unfavorable perceptions of dementia requires policy and public health attention. The results further reveal that an urgent need exists to increase public knowledge about dementia. Social media platforms potentially could be leveraged for future dementia education interventions to increase dementia awareness and promote positive attitudes.

## Introduction

China has the largest population with dementia in the world. An estimated 9.5 million Chinese adults aged 60 years and older had dementia in 2015, accounting for roughly a quarter of the entire population with dementia worldwide [1,2]. Due to China’s aging population, the number of individuals with dementia is projected to reach 23.3 million by 2030 [3]. However, previous studies have found that in China, only around 10% of older people with dementia have a clinical diagnosis, leaving the care needs of a substantial number of families impacted by dementia invisible [4]. Even in the metropolitan regions of China where dementia-related health care services and workforce are available, individuals wait 2 years on average between the onset of symptoms and the first medical visit [5,6]. Such delays are likely more common in underresourced regions and rural China. Furthermore, receiving a clinical diagnosis does not often lead to adequate care. One study reported that over 90% of patients with a dementia diagnosis remain inadequately managed due to a lack of training and knowledge among health care professionals [4,7]. Delayed diagnosis and care impose unnecessary burdens on individuals and family caregivers, as well as increase health care expenditures [8,9]. Extensive evidence indicates that limited knowledge of dementia is a significant predictor of delays in seeking dementia diagnosis and treatment [6,10,11]. An online survey of 10,562 Chinese individuals found that just half of the sample was aware of dementia risk factors [12].

Therefore, improving the public awareness of dementia has been identified as a significant global public health priority. Developing effective approaches to raising the public awareness of dementia requires a solid understanding of current public discourse and attitudes toward the the disease to identify knowledge gaps and priority areas for intervention. However, previous studies in China so far have relied on regional samples or specific stakeholder groups (eg, older adults and their family caregivers [13], mental health providers [14], and health care professionals [15]), with most studies using small samples, thereby providing limited generalizability. As a result, our understanding of dementia awareness, sense making, and comprehension among the Chinese population remains limited.

The phenomenal growth in social media users has provided new avenues for public health research and strategies for health promotion [16]. With 940 million internet users, China reached an internet penetration rate of 67% in 2020 [17]. Obtaining health-related information from social media has become a common practice in China [18]. Not only have platforms such as blogs, online forums, and social networking sites increased patients’ access to information and support, they also have offered a virtual arena in which the general public can express views, opinions, and sentiments about various health conditions. A previous study found that social media has provided a primary avenue through which Chinese people freely share, receive, and circulate knowledge about dementia [19]. Analyzing social media data is a cost-effective and unobtrusive way to assess public perceptions and attitudes toward dementia for 2 reasons. First, compared with survey research, naturally created online user-generated data on social media are less vulnerable to social desirability and recall bias [20]. Using computational tools to analyze social media data concerning dementia may reveal additional information that epidemiological survey research fails to uncover. For instance, social media provides unique sources of information that might be challenging to measure in conventional public health research (eg, personal opinions and experiences), thereby offering new avenues for population health research [21]. Second, utilizing social media data also allows for a more complete understanding of the public’s knowledge and attitudes than previous studies with regional samples, given the high penetration of social media use. Machine learning of user-generated data could identify macrolevel social determinants of population health, which may offer critical insights for public health research, policy, and practice [20].

Weibo (Sina Corporation), founded in 2009, is a Twitter-esque microblogging platform in China. Weibo has 249 million daily active users posting images and short personal stories and perspectives of up to 140 Chinese characters per post. Weibo has grown to be 1 of China’s most prevalent social media platforms. Public opinions expressed on Weibo often reflect current social concerns/sentiments and policy issues in China [22]. Weibo users can follow others, be followed, and create/share/receive information about social events (eg, posting/reposting others’ posts on homepages and broadcasting personal posts to followers), and they can attach photos, videos, universal resource locators (URLs), and emojis to posts [22]. Weibo offers individual (eg, members of the general population, celebrities, and government officials) and organizational accounts that share various information and opinions.

This study analyzed longitudinal user-generated microblogging data from Weibo between January 1, 2010, and July 31, 2021, using machine learning techniques. The study aimed to (1) assess and examine public discourse and sentiment toward dementia among the Chinese public, (2) determine the extent to which dementia-related discourse and sentiment vary among different user groups (ie, government, journalists/news media, scientists/experts, and the general public), and (3) characterize temporal trends in public discourse and sentiment toward dementia among different user groups during the past decade. To the best of our knowledge, this study is the first to examine the public discourse on dementia among Chinese social media users. Findings from this study could inform the development of public health campaigns and interventions aimed at increasing dementia awareness and subsequently improve disease detection and management. Moreover, this study’s findings will contribute to concerted efforts to respond to the World Health Organization’s (WHO) global action plan to raise dementia awareness.

## Methods

### Data Collection and Preprocessing

We used the keyword-based advanced search function provided by Weibo to retrieve relevant data. The selected keywords were informed by sources that included existing studies on media/social media discussions of Chinese dementia/Alzheimer disease [23] and a careful review of 294 Weibo forums (#daily routine of old age idiotic# and #age idiotic# among others) and other social media forums (ie, Baidu Tieba, Zhihu) concerning dementia and Alzheimer disease by 2 authors. Combinations of dementia-related keywords, including “senile dementia” (“老年痴呆” in Chinese), “dementia” (“失智症”), “Alzheimer” (“阿茲海默症”), “brain atrophy” (“脑萎缩”), and their variations (eg, “痴呆症,” “认知障碍,” “小脑萎缩,” “脑梗,” “失智症,” “脑退化,” “阿尔茨海默,” “认知功能障碍”), were selected to retrieve relevant posts between January 1, 2010 (4 months after Weibo was launched in China), and July 20, 2021. After removing duplicates, 983,039 original posts (posts that started/initiated the thread, also known as thread posts; see Multimedia Appendix 1, Figure S1) published by 347,599 unique Weibo users were obtained for analysis. Furthermore, user information (eg, screen name, introduction, user-type status, verification status, follower number, following number, geolocation, education, marital status, gender, registration date, and other information) was also retrieved. Before the subsequent data analysis procedures occurred (ie, latent Dirichlet allocation [LDA] topic modeling, sentiment analysis, and semantic network analyses), we conducted text preprocessing [24] to clean the data (see Multimedia Appendix 1, “Text Preprocessing Procedures,” for detailed preprocessing procedures).

### Data Analysis

#### Dementia-Related Themes

We conducted topic modeling using LDA to identify dementia-related themes. Topic modeling is a computer-assisted content analysis machine learning technique that is semiautomated and unsupervised [25-29]. It is a form of semantic analysis in which statistical algorithms are used to identify abstract or hidden themes that arise from a large corpus of text. This approach presumes that each collection of documents has a given number of hidden themes and that terms associated with a certain subject are used commonly in close proximity to one another. LDA, 1 of the most widely used topic-modeling techniques, classifies texts into latent topics, each of which is represented by an extracted cluster of keywords based on the computed probability of keyword co-occurrence [30].

Based on the results of the LDA modeling process, we chose the optimal and highly granular k value of 30 topics (for details, see Multimedia Appendix 1, “Interpreted Topics With Top 10 Words and Associated Frames”). Specifically, we used statistical indicators of model fit to determine the optimal number of topics, during which a range of models containing 10-300 topics were estimated. Models were examined using skips of 10 for 10-100 topics, skips of 20 for 101-200 topics, and skips of 50 for 201-300 topics. Next, a smaller range of k-topics was selected based on 4 different model fit indicators. We chose the k areas in which indicators of accuracy [31] and density [32] were minimized, and indicators from latent concept modeling and a Bayesian Markov chain Monte Carlo algorithm were maximized [33,34]. For the chosen limited range, we reran the models without skips (eg, after the first iteration pointed to the range of 30-40, we modeled each of the possible 11 models for this corpus). Next, we examined 3 types of information to interpret and label topics and extract potential themes among them, including words with the highest loading on each topic, words that were both prevalent in and exclusive to each topic, and full Weibo posts that were most representative of each topic. Four coauthors, including a public health communication specialist, read and labeled the topics. Next, 3 coauthors independently extracted themes using open coding. Preliminary themes were discussed, and a final set of themes was reached via consensus building. This approach is consistent with the assumption that LDA-generated subjects may be recoded or regrouped into meaningful, condensed frames based on conceptual similarity [35].

#### User Groups

Users were classified into 1 of 4 types based on user information supplied and verified by Weibo (see Table 1 for a detailed description of the user groups): (1) government, including all levels and departments of the Chinese government; (2) journalists/news media, including newspapers, broadcasting companies, and news websites, as well as their affiliated personnel (eg, journalists, editors, and hosts/hostesses); (3) scientists/experts, including influential individuals and organizations in bioscience, medicine, neuroscience, and related fields (eg, scientists/researchers, research departments, and nongovernment organizations in the related field); and (4) users in the general public who were not included in the first 3 categories. Due to the Weibo data set’s longitudinal nature (2010-2021), we performed analyses to determine temporal changes in public discourse and sentiment across different user groups.

**Table 1:** User group typologies.

User type	Description	Examples	Users (N=347,599), n (%)	Posts (N=983,039), n (%)
Government	Different levels and departments of the Chinese government	Gansu Higher People’s Court, the Publicity Department of the Central Committee of the Communist Party of China (CPC), the Tianjin Public Security Bureau	11,829 (3.40)	70,171 (7.14)
Scientists/experts	Users with expertise in neuroscience, including scientists/researchers in the related field, health care providers, and medical experts	Cao Pusheng, director of oncology and surgeon at the Nanning Hospital of Integrated Traditional Chinese and Western Medicine, and Yang Jian, chief physician of the Department of Neurology, Capital Institute of Pediatrics	24,601 (7.08)	140,924 (14.34)
Journalists/news Media	Journalists and news media, including newspapers, broadcasters, and news websites	*Beijing Youth Daily*, *National Business Daily*	12,138 (3.49)	92,030 (9.36)
General public	Users outside the first 3 groups or without specific identities	Lin Yinwei	299,031 (86.03)	679,914 (69.16)

### Semantic Analysis

We conducted semantic network analysis to cross-validate the results from topic modeling. Specifically, public perceptions of dementia among Chinese netizens were characterized by analyzing the semantic networks of dementia discussions on Weibo among various user groups (eg, government, scientists/experts, journalists/news media, and the general public). Following previous studies’ practices [36-38], this study adopted the semantic network analysis method, an analytical approach for deriving meaning from text. Microblogs were analyzed to identify grammatical structures and relationships between individual words [37]. The semantic networks of dementia discussions on Weibo among different types of users (ie, government, scientists/experts, journalists/news media, and the general public) were analyzed using the ForceAtlas2 layout configuration embedded in Gephi. The top 100 words by frequency in each corpus were retained for concise visualization and comprehension. Keywords used to search for data were removed from the final networks because predominant words are highly likely to link all the other words, which may distort the results [36].

### Sentiment Analysis

Sentiment analysis was conducted to ascertain the positive/negative emotions or feelings conveyed in the dementia-related posts. Sentiments were analyzed by using 2 emotion dictionaries (the *Chinese DLUT-Emotion Ontology* developed by the Dalian University of Technology’s Information Retrieval Laboratory and the *Chinese Emotion Valence Dictionary*) in conjunction with dictionary-based techniques [39-41]. For each post, the algorithm creates a continuous sentiment score ranging from negative to positive, with the number’s magnitude indicating sentiment strength. We further assessed whether each sentiment was associated with user types and post characteristics (eg, publication time and dementia-related themes) in regression models.

## Results

### Top Words in Public Discourse Concerning Dementia

**Figure 1:**
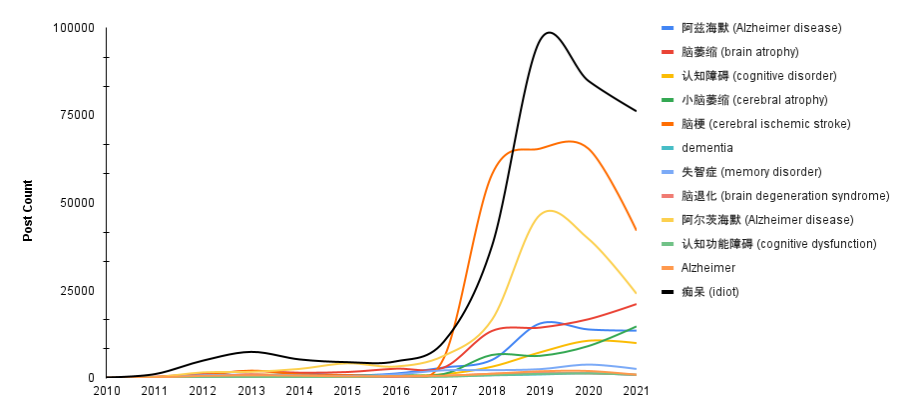
Temporal distribution of search terms in collected posts. Note: “阿兹海默” and “阿尔兹海默” are 2 terms for Alzheimer disease used in simplified Chinese. “脑萎缩,” “认知障碍,” “小脑萎缩,” “脑梗,” “失智症,” “脑退化,” and “认知功能障碍” are synonyms/variations of Alzheimer disease, and 2 terms, including “老年痴呆” (“old age idiotic”) and “痴呆症” (“idiotic disease”) were merged into “痴呆” to display.

**Figure 2:**
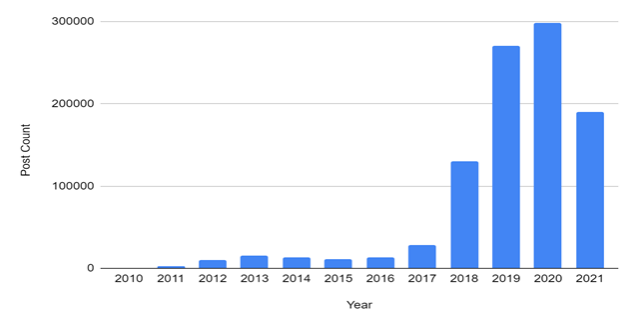
Temporal distribution of dementia-related posts on Weibo. Note: As a result of the sudden drop in data volume in 2021, the year’s data only cover January 1-July 17.

Figure 1 displays the occurrence of each search term over 12 years. Some keywords, including “痴呆” (“stupid” or “idiotic”) and “Alzheimer” (“阿尔兹海默”), occurred more often than others, such as “Alzheimer” (“阿兹海默”), “brain atrophy” (“脑萎缩”), “cognitive impairment” (“认知障碍”), “cerebral atrophy” (“小脑萎缩”), “cerebral infarction” (“脑梗”), “dementia” (“失智症”), “Alzheimer disease” (“脑退化”), and “cognitive dysfunction” (“认知功能障碍”). Figure 2 depicts the temporal distribution of dementia-related posts on Weibo from 2010 to 2021. Since 2018, dementia posts increased significantly.

### Dementia Topics and Themes

We derived 30 latent topics related to dementia from the posts by evaluating the 983,039 dementia-related Weibo posts and reviewing prior research on social media discussions of dementia [42-46]. Table 2 provides the 30 topics and top 10 unique keywords for each topic (a native Chinese-speaking researcher translated the words into English). As illustrated in Table 2, 8 main dementia-related themes were identified, namely social support (n=143,505, 14.60%), advocacy and policy (n=55,905, 5.69%), prevention (n=213,453, 21.71%), media coverage (n=114,949, 11.69%), symptoms (n=246,124, 25.04%), treatment (n=90,959, 9.25%), personal experience (n=87,012, 8.85%), and raising awareness (n=31,132, 3.17%); see Multimedia Appendix 1, “Interpreted Topics With Top 10 Words and Associated Frames,” for frame examples.

**Table 2:** Frames identified from the 30 dementia-related topics on Weibo.

Frame	Posts (N=983,039), n (%)	Description	Subtopics	Representative keywords
Social support	143,505 (14.60)	Support received from formal and informal sources	0, 7, and 10	mom, grandma, take care, children, grandpa, hospital, living, dad, go home, hospitalization, parents, passed away, father, repost/forward, good men/good Samaritan, hope, love, mother, Waterdrop Inc., help, please, life, family, home, elderly/older people, suffering
Advocacy and policy	55,905 (5.69)	Discussions related to policy issues/changes and advocacy initiatives	1, 2, and 21	China, work, overturn, Bailao Yang, human right, street, income, job, service, project, activity, community, society, center, provide, concern, country, medical treatment, free, people with disability, hard-working, temporary worker, Disabled Persons Federation, civil affairs, alone, dismissal, homeless
Prevention	213,453 (21.71)	Preventive biological and lifestyle behaviors	3, 5, 8, 16, 18, 20, 23, 26, and 27	prevention, resolve toxin, brain cells, weight loss, detoxify, suppression, antiaging, protection, effect, pitaya, memory, relaxing bowels, risk, increase, incur, cause, hospitalization, decrease, food, health, cardiovascular, senescence, disease, strengthen, chocolate, emotion, exercise, improve, diabetes, efficacy, enhance, immunity, contain, prevention, stay up all night, prevent, live to the old age, exercise/movement, training, remind, share, expert, blood vessels, sleep, diet, function, reduce, senile dementia/Alzheimer disease, long-term
Media coverage	114,949 (11.69)	Description of dementia on media outlets	4, 9, 13, and 24	news, Weibo, video, driver, recent, doctor, life, bus, send to hospital, one, passenger, an older person, patient, situation, emergency, older persons, being lost, find, older spouse, repost/forward, family, go home, search, tracing notice, phone, search people, help, wear, height, older people, suffering, policeman, one, family members, senile dementia, found, report to police, police station, son, home, be informed of, getting lost, gender, movie, time, father, story, world, trapped, U.S., diagnosis, Alzheimer disease, pass away
Symptoms	246,124 (25.04)	Discussion of different manifestations of dementia	11, 14, 15, 17, 22, and 29	forget, remember, brain, thing, memory, suspicion, ability to remember, a little, omen, patients, cause, diagnosis, decrease, function, usually, myocardial infarction, blood vessel, high blood pressure, stroke, disease, thrombosis, cerebral hemorrhage, blood pressure, heart disease, diabetes, garbage, fat, cardiovascular, blood lipids, find, morning, feeling, come back, go home, go out, forget, symptoms, result in, cognition, performance, influence, neuro, decline, atrophy, dizziness, insomnia, cerebral thrombosis, spinal disease, headache, hand numbness, head, qi and blood, cervical spine, meridian, neck, relief, comfort, nausea
Treatment	90,959 (9.25)	Potential treatment options	6 and 19	treatment, brain atrophy, patient, symptom, cerebral infarction, hospital, doctor, check, disorder, walk, limbs, state of illness, condition, rehabilitation, recovery, traditional Chinese medicine, body, talk, drug, disease, global clinical, protein, neuro, cognitive, new drug
Personal experience	87,012 (8.85)	Sharing individual/family experiences with dementia	12 and 28	older people, suffering, forget, memory, grandma, remember, restaurant, unforgettable, daughter, mother, grandpa, young, cognition, life, barrier, time, one type, cognitive impairment/disorder, understanding, emotion, ability, world, society, self, think, things, change, like, way, learn
Raising awareness	31,132 (3.17)	Posts focusing on fostering greater understanding, attention, and positive attitudes toward dementia	25	Alzheimer disease, patient, World Alzheimer’s Day, China, older people, health, memory, dementia, elderly, prevention, senile, cognition, disease, our country, treatment

### Temporal Distribution of Frames

**Figure 3:**
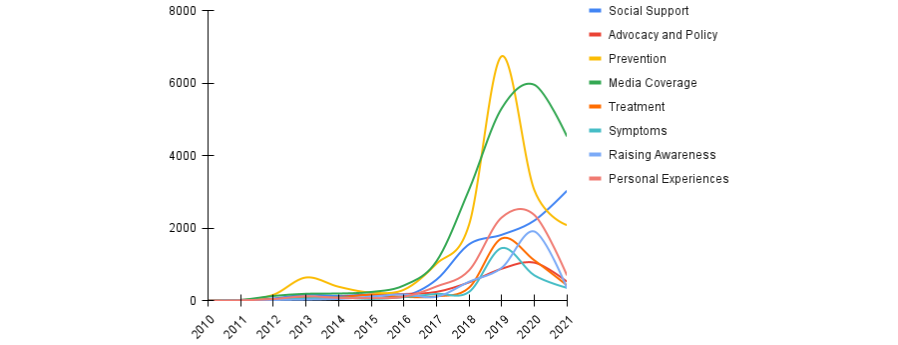
Temporal distribution of dementia-related post frames.

As shown in Figure 3, symptoms, prevention, and social support are the most prevalent dementia frames, while advocacy and policy have been gaining obvious prominence since 2019. Posts about raising awareness of dementia have consistently remained the least used dementia theme in the past decade.

### Theme–User Group Analysis

To examine possible differences in dementia frames across different user groups, we first conducted an 8 (frames) × 4 (user groups) chi-square test, which revealed that dementia frame adoptions significantly differed across the 4 types of users (*χ*^2^_21_=147,217.420, *P*<.001). Specifically, as shown in Table 3, prevention (n=46,437, 32.95%), symptoms (n=30,238, 21.46%), and treatment (n=31,137, 22.09%) frames were relatively salient, with a high proportion for scientists/experts. With regard to the government, media coverage (n=21,133, 30.12%), prevention (n=16,707, 23.81%), and social support (n=9853, 14.04%) were the most commonly used frames. For journalists/news media users, media coverage (n=23,041, 25.04%), prevention (n=19,264, 20.93%), and social support (n=16,267, 17.67) ranked much higher than the other 5 dementia frames. With regard to the general public, treatment (n=216,321, 31.82%), prevention (n=131,978, 19.41%), and social support (n=111,431, 16.39%) were the most frequently adopted frames, followed by media coverage (n=65,167, 9.73%), personal experience (n=62,865, 9.58%), symptoms (n=45,208, 6.76%), advocacy/policy (n=43,369, 6.78%), and raising awareness (n=18, 0.003%). Overall, for dementia framing, the general public is more aligned with scientists/experts, while the government and journalists/news media users share a similarity.

**Table 3:** Differences in dementia frames among different user groups.

Function	Government (n=70,171), n (%)	Scientists/experts (n=140,924), n (%)	Journalists/newsmedia (n=92,030), n (%)	General public (n=679,914), n (%)
Social support	9853 (14.04)	6494 (4.61)	16,267 (17.67)	111,431 (16.39)
Advocacy and policy	3788 (5.40)	4233 (3.00)	4250 (4.62)	46,132 (6.78)
Prevention	16,707 (23.81)	46,437 (32.95)	19,264 (20.93)	131,978 (19.41)
Media coverage	21,133 (30.12)	4514 (3.20)	23,041 (25.04)	65,167 (9.58)
Symptoms	4206 (6.00)	30,238 (21.46)	10,303 (11.20)	45,988 (6.76)
Treatment	3285 (4.68)	31,137 (22.09)	5909 (6.42)	216,321 (31.82)
Personal experience	7020 (10.00)	7140 (5.07)	8888 (9.66)	62,865 (9.25)
Raising awareness	4179 (5.95)	10,731 (7.61)	4108 (4.46)	32 (0.003)

### Themes in Public Discourse Concerning Dementia

Table 4 presents the clusters and top word associations, as well as the percentage share of the network for each subcluster. We identified 5 word clusters in the government semantic network. Among them, cluster 3 (33.33% of the semantic network) was the most prevalent frame, focusing on prevention, followed by cluster 2 (25.00%) on social support; cluster 5 (20.83%) on symptoms; cluster 1 (14.58%) on media coverage, treatment, and social support; and cluster 4 (6.25%) on personal experience. For scientists/experts, 4 clusters of words were identified: cluster 2 (30.53%) and cluster 3 (23.16%) focusing mostly on prevention, cluster 4 (24.21%) on treatment, and cluster 1 (22.11%) on symptoms. For journalists/news media, media coverage emerged as the most prevalent word cluster (37.76%), followed by personal experience (ie, cluster 2: 27.55%), social support (ie, cluster 4: 23.47%), and symptoms (ie, cluster 1: 11.22%). In terms of the general public’s semantic network, personal experience (ie, cluster 4: 64.65% of the semantic network) was the dominant cluster, followed by social support (ie, clusters 1, 2, and 3: 10.10%, 16.16%, and 9.1% of the semantic network, respectively).

**Figure 4:**
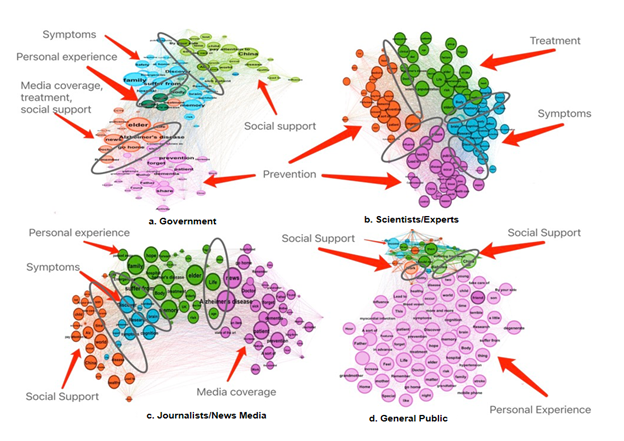
Semantic network visualization of dementia-related discussions among different users on Weibo. Note: All words are in lowercase.

As shown in Figure 4, the distance between 2 clusters indicates their coshared words’ proportion and their association in the semantic network. For the government, prevention was closer to media coverage, treatment, and social support, although symptoms shared more keywords with personal experience and social support. For scientists/experts, prevention had more connections with treatment and symptoms. For journalists/news media, personal experiences were related more to media coverage and symptoms, while symptoms also shared more similarities with social support. For the general public, the most prominent cluster association was between personal experiences and social support.

**Table 4:** Summary results of network modularity analysis of semantic networks by 4 user groups.

Network and clusters			Associated frames		Top terms		Share of network, (%)
**Government**							
	1	Media coverage, treatment, social support		CCTV, elder, news, go home, life, doctor, remember, treatment, policeman, ask for help		14.58%	
	2	Social support		pay attention to, son, disease, child, by your side, take care of, lead to, healthy, parents, spouse		25.00%	
	3	Prevention		prevention, forget, sick, father, mother, suffer from, patience, activity, life, increase		33.33%	
	4	Personal experience		disease, hope, body, children, chronic, forward		6.25%	
	5	Symptoms		suffer from, memory, discover, hospital, brain, safety, research, emergencies, risk, cognition, personal		20.83%	
**Scientists/experts**							
	1	Symptoms		brain, memory, research, body, discover, cognition, early, symptoms, hospital, effects, dysfunction		22.11%	
	2	Prevention		reduce, publish, lead to, healthy, article, health care, blood, drug, expert, control, cardiovascular, blood vessel, diabetes		30.53%	
	3	Prevention		improve, prevention, patient, medicine, food, performance, increase, features, long-term		23.16%	
	4	Treatment		elderly, risk, treatment, life, science popularization, doctor, stroke, factors, hypertension, diet		24.21%	
**Journalists/news media**							
	1	Symptoms		discover, brain, cognition, personnel, sleep, worldwide, publish, symptoms, expert, drug		11.22%	
	2	Personal experience		suffer from, elder, family, memory, life, hope, body, treatment, hospital, emergencies, risk		27.55%	
	3	Media coverage		news, patient, doctor, prevention, forget, go home, sick, state-of-art, father, remember, work, increase		37.76%	
	4	Social support		work, healthy, China, time, disease, child, son, lead to, pay attention to, occur, take care of, by your side		23.47%	
**General public**							
	1	Social support		work, disabled, disabled people’s federation, child union, China Unicom, sweat, more than 30 years, repay debt, get back, flaunt, Kuomintang		10.10%	
	2	Social support		diabetes, China, suffer from, street, since ancient times, overthrow, temporary worker, superiority, homeless, dismissal, kill for life		16.16%	
	3	Social support		income, nowadays, human rights, alone, civil affair, hard-earned money, tens of millions, fabricated		9.1%	
	4	Personal experience		life, father, matter, elder, memory, mother, discover, degenerate, home, life, hope, mother		64.65%	

### Sentiment Toward Dementia

Results from the sentiment analyses are presented in Table 5. The findings revealed that dementia-related Weibo posts published in 2011 (B=2.85, *P*=.002), 2012 (B=2.64, *P*=.002), and 2016 (B=1.69, *P*=.05) were significantly more positive compared with those published in 2010 (see Multimedia Appendix 1, “Sentiment Analysis Results by Year, Frame, and User Type,” for sentiment mean values for each year). In terms of user groups, government users (B=5.81, *P*<.001), scientists/experts (B=3.22, *P*<.001), and journalists/news media (B=5.11, *P*<.001) tended to be more positive than the general public concerning dementia discourse on Weibo. Moreover, when compared to personal experiences, only posts on advocacy and policy were associated with positive sentiments (B=3.16, *P*<.001), whereas posts on social support (B=–5.27, *P*<.001), prevention (B=–3.17, *P*<.001), media coverage (B=–3.52, *P*<.001), symptoms (B=–3.55, *P*<.001), treatment (B=–9.49, *P*<.001), and raising awareness (B=–4.34, *P*<.001) tended to express more negative emotions.

**Table 5:** Regression model predicting sentiment with dementia-related messages on Weibo (R^2^=6%, N=983,039).

Year, frame, and user type		Estimate	95% CI	*P* value^a^
**Dementia-related frames (reference group=personal experience)**				
	Social support	–5.27	–5.42 to –5.12	*<.001* ^b^
	Advocacy and policy	3.16	2.97-3.35	*<.001* ^b^
	Prevention	–3.17	–3.31 to –3.04	*<.001* ^b^
	Media coverage	–3.52	–3.67 to –3.36	*<.001* ^b^
	Symptoms	–3.55	–3.71 to –3.38	*<.001* ^b^
	Treatment	–9.49	–9.63 to –9.36	*<.001* ^b^
	Raising awareness	–4.34	–4.57 to –4.11	*<.001* ^b^
**User groups (reference group=general public)**				
	Government	5.81	5.67-5.95	*<.001* ^b^
	Scientists/experts	3.22	3.11-3.32	*<.001* ^b^
	Journalists/news media	5.11	4.98-5.23	*<.001* ^b^
**Year (reference group=2010)**				
	2011	2.85	1.04-4.66	*.002* ^c^
	2012	2.64	0.94-4.34	*.002* ^c^
	2013	1.56	–0.13 to 3.24	.07
	2014	0.54	–1.15 to 2.23	.53
	2015	1.29	–0.40 to 2.99	.14
	2016	1.69	0.00-3.38	*.05* ^d^
	2017	0.51	–1.17 to 2.18	.55
	2018	1.42	–0.24 to 3.09	.09
	2019	0.48	–1.18 to 2.15	.57
	2020	–0.46	–2.13 to 1.20	.59
	2021	0.52	–1.15 to 2.18	.54
	Constant	7.63	5.96-9.30	.01^d^

^a^Italicized *P* values are significant.

^b^*P*<.001.

^c^*P*<.01.

^d^*P*<.05.

## Discussion

### Principal Findings

By analyzing 983,039 Weibo posts from January 1, 2010, to July 20, 2021, using machine learning techniques, this study discovered prominent themes and feelings tied to dementia among the Chinese population, as well as noteworthy temporal trends over the past decade. Specifically, between 2018 and 2021, dementia garnered an increasing amount of public attention on social media. In particular, exponential growth in dementia-related posts has been occurring since 2018. Several explanations for this temporal shift are possible. First, the drastic rise in public attention being paid to dementia could be attributed partly to the celebrity effect. For instance, Sir Charles Kuen Kao, the father of fiber optics and recipient of the Nobel Prize in Physics, died in September 2018 following a decade-long battle with Alzheimer disease. Kao and his family established the Charles K. Kao Foundation for Alzheimer’s Disease in 2010 to raise public awareness about Alzheimer disease and dementia in Chinese society. The Kao family’s public disclosure received extensive coverage in Chinese media, which may have led to the growing trend since 2018. Indeed, the results from the time series analysis using the time of Sir Kao’s death as the time of event indicated significant changes in monthly dementia post counts (see Multimedia Appendix 1, “Interrupted Time Series,” for detailed results). Similarly, a reality show “Forget Me Not Café,” starring 5 older people with dementia, premiered in China in 2019 to raise public awareness about dementia. The show received around 6.7 million views between its debut and February 2021 [47]. Second, a succession of dementia-related initiatives in China also may have contributed to increased discussion of dementia on social media [10,48]. For instance, Alzheimer’s Disease China (ADC) was founded in 2002 to serve as the Alzheimer’s Disease International’s Chinese affiliate. In 2011, China’s 12th Five-Year Development Plan for Population Aging recognized the need for early dementia intervention. In 2014, the first Memory Clinic Guide was published to aid in the timely diagnosis and treatment of dementia. In 2018, about 100 memory clinics were in operation. Furthermore, the National Health Commission of the People’s Republic of China launched the *Alzheimer’s Disease Prevention and Treatment Guide* in 2019 [49]. Collectively, these national initiatives may have contributed greatly to an upsurge in dementia conversations on social media.

Our results indicate that the derogatory term for dementia in China, “老年痴呆,” which roughly translates to “old and stupid,” has remained the most frequently used term for dementia on social media over the past decade. Although alternative terms for dementia have been introduced and advocated in China [50], this derogatory term continues to dominate public discourse. Considering that existing research suggests that derogatory local terms for dementia may result in strong stigmatization and, thus, discourage people from seeking help, multiple East Asian nations (eg, Japan, Hong Kong, and Singapore) have launched campaigns to replace their negative local terms for dementia with more neutral biomedical ones [51], such as “cognitive disorder” (“認知障礙症”) and “brain degeneration syndrome” (“腦退化症”) in Hong Kong and “memory disorder” (“失智症”) in Singapore and Taiwan. A national effort to rename dementia terms is essential in helping to change public views and attitudes toward dementia in Mainland China. Nevertheless, it is worth noting that advocacy and policy have been gaining momentum on Chinese social media since 2019. Collectively, these results indicate that although dementia has been linked with stigmatizing local terms, the future looks bright, as advocacy- and policy-related discussions gain momentum.

Another noteworthy finding is that raising awareness (ie, posts promoting greater understanding and positive attitudes toward dementia) has remained the least discussed dementia-related subject on social media. For example, a Weibo user published a post that read, “To appeal to society, many elderly people in rural areas are not simply old and confused; they are just sick,” highlighting the need to eradicate prejudice toward older adults with dementia. This is concerning, given the overwhelming evidence supporting the importance of raising awareness to promote early detection and treatment of dementia [10,52]. These findings underscore the urgent need for public health campaigns on dementia in China. Improved awareness and knowledge of dementia may help reduce stigmatization and discrimination, as well as enhance social support for affected individuals and their families [53,54], thereby helping to develop dementia-friendly communities in which families affected by dementia are supported and included [55]. Given the surge in social media use, leveraging social media outlets offers a cost-effective way to raise public awareness of dementia (eg, sharing dementia experiences, stories, and knowledge via public messaging and general communication). In some countries (eg, Australia), media campaigns have been critical in gaining government recognition of dementia as a national health priority [53].

The consistent results from 2 typical text-mining methods (ie, topic modeling and semantic network analysis) indicate our findings’ robustness. Our findings suggest that symptoms, treatment, and prevention, which often indicate deficits and incapacities, are the most discussed aspects of dementia overall. The dominance of such medical discourse may exacerbate stigmatization and fear toward those with dementia [56,57]. Semantic analysis further revealed that various Weibo user groups focus on distinct aspects of dementia. Specifically, government users focused primarily on prevention, social support, and symptom management, while prevention, treatment, and symptoms dominated scientists/experts’ discourse. Media coverage, personal experiences, and social support dominated journalists/news media’s online discourse, while personal experience and social support aspects were relatively more prominent in the general public’s discourse. Furthermore, dementia frame adoption differed significantly across the 4 user groups. Government users and journalists/news media users had a greater degree of agreement on dementia discourse, with a particular emphasis on media coverage, prevention, and social support. However, the general public and scientists/experts mostly emphasized treatment, prevention, and social support.

Regarding sentiment toward dementia, our findings suggest that the general public is more prone than other user groups (ie, the government, journalists/news media, and scientists/experts) toward harboring negative sentiments toward dementia. Furthermore, compared with posts on personal experiences, only microblogging posts with a policy and advocacy focus indicated positive sentiments. All the remaining dementia frames/themes were associated with negative sentiments. Furthermore, an interesting temporal trend was discovered. Specifically, our results reveal that compared with the baseline in 2010, sentiments toward dementia on Chinese social media were more positive from 2011 to 2017. Collectively, sentiment analyses indicate that even though sentiment toward dementia has become more positive over the years, it remains negative overall, particularly among the general public. Owing to the availability of advanced analytical tools, these findings add an emotional dimension to our understanding of public opinion on dementia in China.

Furthermore, this study’s results further reveal that a variety of user groups (eg, the general public, health care providers, and policymakers) utilize Weibo to receive and share information related to dementia. This finding corresponds with earlier research indicating that social media platforms are the primary information-seeking and information-sharing medium for dementia discourse [19,42]. Consequently, Weibo could serve as a medium for dementia awareness-raising and educational initiatives.

### Implications

The study’s findings carry significant implications for research, policy, and practice. First, considering diverse user groups’ active engagement and the abundance of microblogging content concerning dementia, Weibo could be leveraged as a primary channel through which to disseminate dementia-related educational programs to promote awareness and positive attitudes toward dementia. Future research needs to evaluate whether Weibo could be an effective modality for engaging the general public in dementia education and other health promotion activities related to dementia. Second, despite national policy and awareness-raising campaigns, the most widely used Chinese term for dementia is still one that carries considerable negative connotations. Thus, a renaming campaign is needed urgently in Mainland China. Third, the results reveal widespread unfavorable attitudes toward dementia, particularly among the general public, highlighting the critical need for public health campaigns with multiprong approaches (eg, academic institutions collaborating with community organizations and health care delivery organizations, including hospitals and clinics, to produce more dementia-friendly films, documentaries, reality shows, and educational brochures). Furthermore, computational social science is gaining prominence. Advanced information processing analytical tools (eg, machine learning) are being used to analyze vast amounts of data to acquire a better understanding of social and public health issues. Future studies need to deepen similar public health–computational social science collaborations to advance our knowledge of a variety of social challenges. Moreover, our findings reveal that dementia is connected constantly with negative sentiments, with symptoms and prevention dominating public discourse. Developing interventions to combat such an overtly unfavorable view of dementia requires policy and public health attention. Finally, although keywords used in this study are in line with common practices in the field, new terms or words that refer to dementia may emerge on social media as online discourses may evolve over time. Future researchers can build on our keyword list and actively monitor and add new keywords to develop an enhanced and dynamic understanding of dementia (eg, leveraging “keywords-as-frames” as the discourse analysis tool in future studies).

### Limitations and Strengths

Several limitations of the study warrant discussion. First, considering that the data used in this study were obtained only from Weibo users, the study’s findings may not be generalizable to the entire Mainland China’s population (eg, those without internet access and non-Weibo users). Certain populations’ voices (eg, those with dementia and their caregivers) may not be represented in this study, owing to possible difficulties with social media navigation. The lived experiences of people with dementia and their family caregivers require further investigation to refute prevailing negative and biomedical discourses about dementia [57]. Moreover, it is possible that some extreme views on dementia and negative sentiments may not be available on these social media platforms; thus, such views may not have been captured in this study. Nevertheless, considering that Weibo is China’s most popular social media site, the study’s findings reflect population-level perceptions of dementia in China. Future studies using large-scale data from other sources are needed to corroborate our results. Furthermore, this study examined the public perception of dementia only in Mainland China; thus, further research into cross-cultural comparisons of social media representations of dementia in other countries/cultures is warranted.

Despite these limitations, this study overcomes other limitations inherent in survey research on dementia, in which realizing a national understanding of public discourse and opinion concerning dementia remains challenging. The findings built on existing research by adding a macrolevel understanding of the Chinese public’s discourse and sentiment toward dementia, which is critical for developing national education and policy initiatives to create a dementia-friendly society. Furthermore, our findings respond to the global call to expand dementia research in low- and middle-income countries [53].

### Conclusion

Dementia-related discussions have expanded rapidly on social media in China during the past decade, most notably since 2018. However, a derogatory local Chinese term for dementia remains in common use; thus, a national renaming effort is imperative to reform the public discourse on dementia. Discussions related to dementia advocacy and policy are gaining momentum in China, but promoting awareness of dementia has remained the least discussed topic over time. The sentiment toward dementia is negative overall in social media, particularly among the general public, but social media platforms are part of a viable medium for providing dementia education and awareness-raising interventions in Mainland China.

## Appendix

Multimedia Appendix 1 Data collection and preprocessing.

## Abbreviations

LDA: latent Dirichlet allocation
