# Supplementary material for: Public Discourse and Sentiment Toward Dementia on Chinese Social Media: Machine Learning Analysis of Weibo Posts
Source: J Med Internet Res. 2022 Sep 2;24(9):e39805. doi: 10.2196/39805 (PMC9482068; doi:10.2196/39805)
Supplement: Multimedia Appendix 1 [file jmir_v24i9e39805_app1.docx]

**Multimedia Appendix 1. Data collection and preprocessing.**

**S1. Illustration of Original Post**


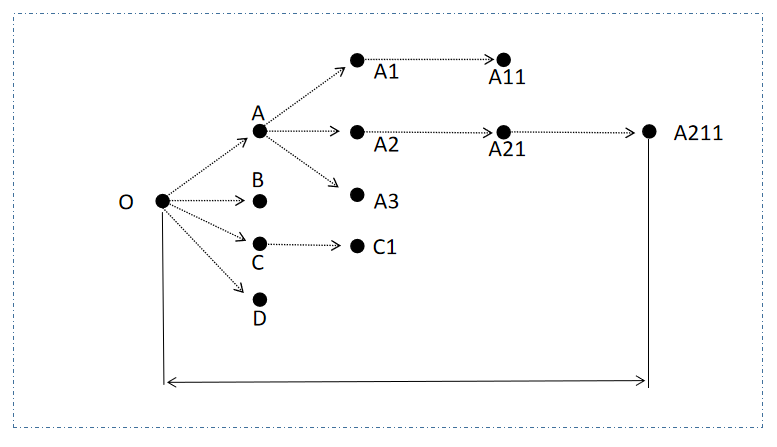


Figure S1. Demonstration of the diffusion thread of a post. In this example, O is the original post

**S2. Text pre-processing procedures**

First, for text pre-processing, punctuation marks were removed, and the Jieba Python toolkit was applied to complete the Chinese word segmentations. Furthermore, several widely used stopword dictionaries––including hit_stopwords (created by the Harbin Institute of Technology, China), baidu_stopwords (created by Baidu, a Chinese company that provides web services, including a search engine with the same name, a leader among Chinese search engines), and scu_stopwords (created by Machine Intelligence Laboratory of Sichuan University, China)––were used to filter out meaningless words (i.e., commonly used words that are meaningless in natural language processing, e.g., “the,” “a,” “an,” and “in”). A tokenization process then was applied with several Python packages (i.e., Pickle, CountVectorizer, and TfidfVectorizer), in which text was split into units with semantic meanings, e.g.:

● Raw text: “I ate a burger, and it was good.”

● Tokenized text: [“I,” “ate,” “a,” “burger,” ,”,” “and,” “it,” “was,” “good,” “.”].

S3. Calculates different metrics to estimate the most preferable number of topics for the LDA model.

**
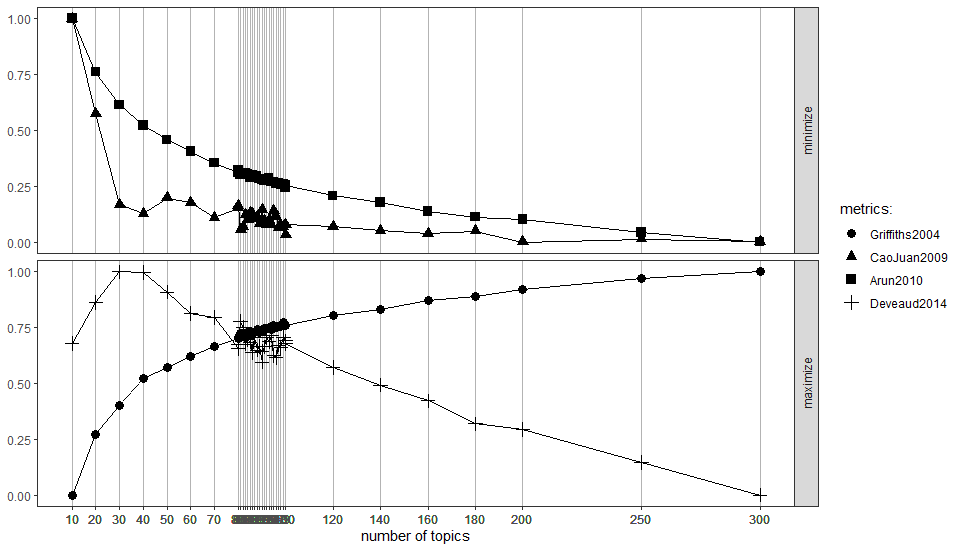
**

**S4. Interpreted Topic with Top 10 words, and Associated Frame.**

| Topic No. | Top Words | N of Post | Topic |
| --- | --- | --- | --- |
| 0 | 妈妈 脑梗 希望 奶奶 照顾 孩子 爷爷 医院 生活 爸爸 家里 身体 回家 住院 父母 看着 去世 好好 两个 不好 | 80,472 | Social Support |
| 1 | 当年 现如今 中国 工作 推翻 杨白劳 现演 杜撰 千万个 国民党 糖尿病 标榜 人权 2018 患脑 2016 街道 收入 不该 | 18,364 | Advocacy and Policy |
| 2 | 工作 服务 项目 活动 社区 社会 医院 成功 中心 提供 关注 国家 医疗 中国 生活 免费 公司 相关 全国 情况 | 28,925 | Advocacy and Policy |
| 3 | 预防 痴呆症 发生 排毒 脑细胞 减肥 皮肤 解毒 抑制 抗衰老 保护 变性 功效 火龙果 记忆力 果实 颜色 润肠 | 12,766 | Prevention |
| 4 | 脑梗 突发 医院 司机 近日 医生 生命 公交 送医 一位 乘客 一名 新闻 老人 患者 情况 紧急 公交车 | 26,284 | Media Coverage |
| 5 | 老年痴呆 睡眠 作业 孩子 时间 长期 风险 研究 增加 引发 大脑 导致 住院 脑梗 辅导 发现 老人 阿尔茨海默 | 20,845 | Prevention |
| 6 | 治疗 脑萎缩 患者 症状 脑梗 医院 医生 检查 失调 走路 肢体 病情 情况 康复 恢复 文章 中医 病人 身体 说话 | 39,786 | Treatment |
| 7 | 脑梗 医院 治疗 父亲 转发 好心人 希望 这是 爱心 母亲 水滴筹 医生 帮帮忙 突发 拜托 生活 住院 家庭 手术 家里 | 30,316 | Social Support |
| 8 | 痴呆症 风险 研究 老年痴呆 降低 食物 健康 心血管 衰老 疾病 大脑 能力 增加 记忆力 退化 老人 发现 增强 巧克力 | 19,588 | Prevention |
| 9 | 老人 走失 阿尔茨海默 患有 老年痴呆 找到 老伴 转发 家人 新闻 回家 寻找 脑萎缩 寻人启事 电话 寻人 帮忙 身穿 身高 | 23,091 | Media Coverage |
| 10 | 老人 母亲 照顾 父亲 患有 老年痴呆 脑萎缩 脑梗 新闻 儿子 发现 一位 突发 一手 女儿 妻子 近日 丈夫 | 32,717 | Social Support |
| 11 | 老年痴呆 感觉 忘记 记得 事情 脑子 东西 提前 越来越 好像 记性 怀疑 记忆 喜欢 名字 不好 记忆力 一点 脑萎缩 前兆 | 80,383 | Symptoms |
| 12 | 阿尔茨海默 老人 阿兹海默症 患有 忘记 记忆 奶奶 记得 餐厅 忘不了 女儿 母亲 一位 痴呆症 爷爷 海默症 年轻 | 47,263 | Personal Experience |
| 13 | 老人 患有 民警 一位 阿尔茨海默 微博 家人 发现 老年痴呆 找到 回家 报警 派出所 儿子 近日 走失 家中 得知 迷路 | 26,233 | Media Coverage |
| 14 | 大脑 记忆 记忆力 患者 导致 患上 疾病 阿尔茨海默 痴呆症 影响 下降 健康 办法 长寿 年轻 功能 平时 | 12,437 | Symptoms |
| 15 | 脑梗 心梗 血管 高血压 健康 中风 血液 疾病 血栓 脑溢血 血压 心脏病 糖尿病 身体 发生 心脏 垃圾 脂肪 心脑血管 血脂 | 34,941 | Symptoms |
| 16 | 老年痴呆 疾病 失智症 演讲 分享 带来 健康 大脑 情绪 保护 抑郁 运动 作用 专注 生命 免受 患上 | 9,883 | Prevention |
| 17 | 晚上 发现 早上 手机 感觉 东西 回来 回家 昨天 出门 忘记 睡觉 下午 不到 家里 提前 小时 上班 脑子 | 69,428 | Symptoms |
| 18 | 预防 老年痴呆 改善 糖尿病 癌症 作用 疾病 功效 人体 高血压 提高 免疫力 中风 心脏病 失眠 血管 健康 记忆力 增强 含有 | 29,329 | Prevention |
| 19 | 阿尔茨海默 研究 治疗 发现 发表 美国 药物 患者 疾病 痴呆症 一种 全球 一项 人员 临床 蛋白 神经 认知 大学 新药 | 51,173 | Treatment |
| 20 | 老年痴呆 预防 熬夜 发表 脑萎缩 发现 文章 阻止 活到老 一篇 健康 锻炼 研究 转载 运动 训练 世界 中国 老年 | 27,563 | Prevention |
| 21 | 工作 糖尿病 中国 患脑 瘫 不该 收入 街道 残疾人 辛辛苦苦 临时工 三十多年 残联 民政 孤身一人 分文 解雇 无家可归 | 8,616 | Advocacy and Policy |
| 22 | 疾病 脑萎缩 功能 治疗 患者 障碍 症状 导致 一种 认知 表现 影响 发生 神经 原因 生活 大脑 系统 下降 萎缩 | 29,727 | Symptoms |
| 23 | 老年痴呆 发生 预防 忘记 海默症 父母 阿尔兹 希望 患病 慢慢 绝症 俗称 身上 阿兹海默症 儿女 慢性 老人 医院 | 19,367 | Prevention |
| 24 | 老年痴呆 认知障碍 视频 微博 性别 电影 时间 父亲 阿兹海默症 阿尔茨海默 故事 世界 困在 一部 美国 剧情 患者 患有 阿兹海默 去世 | 39,341 | Media Coverage |
| 25 | 阿尔茨海默 患者 老年痴呆病日 [世界 阿茨海默病日](https://baike.baidu.com/item/%E4%B8%96%E7%95%8C%E9%98%BF%E5%B0%94%E8%8C%A8%E6%B5%B7%E9%BB%98%E7%97%85%E6%97%A5/19978691) 中国 老人 健康 记忆 老年痴呆症 病患者 老年人 预防 痴呆 认知 疾病 我国 治疗 | 31,132 | Raising awareness |
| 26 | 脑梗 医生 心梗 文章 健康 提醒 分享 专家 食物 疾病 血管 文章 发生 导致 新闻 看点 越来越 情况 | 36,284 | Prevention |
| 27 | 大脑 研究 风险 增加 健康 导致 发现 睡眠 影响 饮食 功能 降低 运动 预防 减少 老年痴呆 长期 生活 疾病 记忆力 | 37,819 | Prevention |
| 28 | 认知 生活 障碍 时间 一种 认知障碍 理解 情绪 能力 事情 人生 世界 社会 自我 思考 东西 改变 喜欢 方式 学习 | 39749 | Personal Experience |
| 29 | 脑梗 头晕 失眠 导致 健康 脑血栓 颈椎病 头痛 头疼 手麻 头部 气血 颈椎 经络 微博 脖子 缓解 舒服 恶心 | 19,208 | Symptoms |
| N |  | 983,039 |  |

**S5. Sentiment Analysis Results by year, frames, and user type**

|  |  |  | 95% Confidence Interval for Mean | |
| --- | --- | --- | --- | --- |
|  | Mean | SD | Lower | Upper |
| **Time** |  |  |  |  |
| 2010 | 5.24 | 8.72 | 4.41 | 6.07 |
| 2011 | 8.49 | 11.32 | 8.03 | 8.96 |
| 2012 | 8.41 | 10.34 | 8.21 | 8.61 |
| 2013 | 7.86 | 10.65 | 7.69 | 8.03 |
| 2014 | 6.77 | 11.40 | 6.58 | 6.97 |
| 2015 | 7.65 | 9.93 | 7.47 | 7.84 |
| 2016 | 7.84 | 14.00 | 7.60 | 8.08 |
| 2017 | 7.15 | 14.96 | 6.98 | 7.33 |
| 2018 | 5.71 | 17.06 | 5.61 | 5.80 |
| 2019 | 4.79 | 18.77 | 4.72 | 4.86 |
| 2020 | 4.00 | 18.34 | 3.93 | 4.07 |
| 2021 | 4.43 | 19.03 | 4.35 | 4.52 |
| **User Type** |  |  |  |  |
| Government | 10.59 | 22.52 | 10.42 | 10.75 |
| Scientist/Experts | 6.85 | 22.19 | 6.73 | 6.96 |
| Journalist/ News Media | 9.75 | 17.87 | 9.64 | 9.87 |
| General Publics | 3.17 | 16.11 | 3.13 | 3.21 |
| **Frame** |  |  |  |  |
| Social Support | 3.90 | 17.59 | 3.81 | 3.99 |
| Advocacy and Policy | 11.92 | 34.26 | 11.64 | 12.20 |
| Prevention | 6.59 | 17.44 | 6.52 | 6.67 |
| Media Coverage | 6.79 | 12.29 | 6.72 | 6.86 |
| Treatment | 6.50 | 22.02 | 6.36 | 6.64 |
| Symptoms | -0.91 | 11.34 | -0.95 | -0.86 |
| Raising Awareness | 6.25 | 12.50 | 6.12 | 6.39 |
| Personal Experiences | 9.27 | 18.97 | 9.15 | 9.40 |

**S6. Interrupted Time Series**

Using the death of Sir Charles Kuen Kao (the “father of fiber optics” and recipient of the Nobel Prize in Physics), as the intervention point (September 2018), we conducted the time series interrupted analysis as shown in Figure 1 and Table 1. The results showed significant changes in monthly dementia post count. The coefficients in Table 1 could be interpreted as follow:

- The Time coefficient indicates the monthly dementia post count trend before the intervention. It’s positive and significant, indicating that monthly dementia post count increases over time. For each month that passes, the monthly dementia post count increases by 113.53 points on the index.
- The Treatment coefficient indicates the increase in the monthly dementia post count immediately after the intervention. We can see that the immediate effect is positive and significant, indicating that the death of Sir Charles Kuen Kao increased the monthly dementia post count of 17,673.45.
- The Time Since Treatment coefficient indicates that the trend has changed after the intervention. The sustained effect is negative and significant, indicating that for each month that passes after the intervention, the monthly dementia post count decreases of 511.11 points on the index.
- Though the monthly dementia post count drastically decreased after the month of Sir Charles Kuen Kao’s death, it remains at a much higher level, compared to the pre-intervention periods.

​​
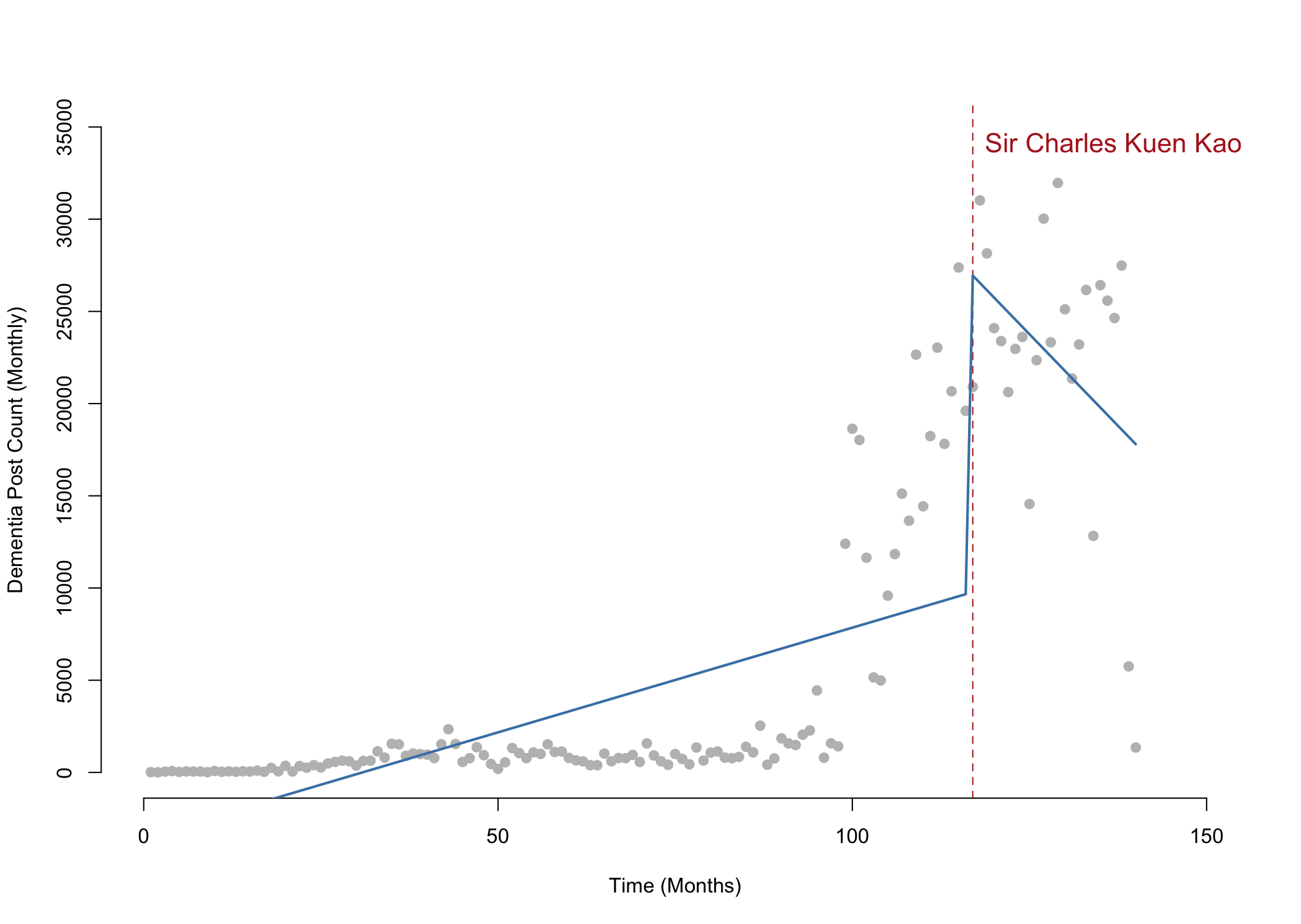


Figure 1. Time series interrupted analysis results visualization

|  | Dependent variable: |
| --- | --- |
|  | Dementia Post Count (Monthly) |
|  | Model results |
| Time | 113.53***  (13.98) |
| Treatment | 17,673.45***  (2,318.31) |
| Time Since Treatment | -511.11***  (149.28) |
| Constant | -3,503.14*** |

Note: *p<0.1; **p<0.05; ***p<0.01
